# Changes in EEG Complexity with Electroconvulsive Therapy in a Patient with Autism Spectrum Disorders: A Multiscale Entropy Approach

**DOI:** 10.3389/fnhum.2015.00106

**Published:** 2015-02-26

**Authors:** Ryoko Okazaki, Tetsuya Takahashi, Kanji Ueno, Koichi Takahashi, Makoto Ishitobi, Mitsuru Kikuchi, Masato Higashima, Yuji Wada

**Affiliations:** ^1^Department of Neuropsychiatry, Faculty of Medical Sciences, University of Fukui, Fukui, Japan; ^2^Department of Child and Adolescent Mental Health, National Center of Neurology and Psychiatry, National Institute of Mental Health, Tokyo, Japan; ^3^Research Center for Child Mental Development, Kanazawa University, Kanazawa, Japan

**Keywords:** autism spectrum disorders, brain-derived neurotrophic factor, EEG complexity, electroconvulsive therapy, multiscale entropy, obsessive–compulsive disorder

## Abstract

Autism spectrum disorders (ASD) are heterogeneous neurodevelopmental disorders that are reportedly characterized by aberrant neural networks. Recently developed multiscale entropy analysis (MSE) can characterize the complexity inherent in electroencephalography (EEG) dynamics over multiple temporal scales in the dynamics of neural networks. We encountered an 18-year-old man with ASD whose refractory catatonic obsessive–compulsive symptoms were improved dramatically after electroconvulsive therapy (ECT). In this clinical case study, we strove to clarify the neurophysiological mechanism of ECT in ASD by assessing EEG complexity using MSE. Along with ECT, the frontocentral region showed decreased EEG complexity at higher temporal scales, whereas the occipital region expressed an increase at lower temporal scales. Furthermore, these changes were associated with clinical improvement associated with the elevation of brain-derived neurotrophic factor, which is a molecular hypothesis of ECT, playing key roles in ASD pathogenesis. Changes in EEG complexity in a region-specific and temporal scale-specific manner that we found might reflect atypical EEG dynamics in ASD. Although MSE is not a direct approach to measuring neural connectivity and the results are from only a single case, they might reflect specific aberrant neural network activity and the therapeutic neurophysiological mechanism of ECT in ASD.

## Introduction

Autism spectrum disorders (ASD) are heterogeneous neurodevelopmental disorders that are complicated by the co-existence of and symptomatic overlap with other psychiatric disorders (Leyfer et al., 2006). Among other factors, the frequent presence of obsessive–compulsive symptoms in ASD has been emphasized in earlier study (Mack et al., 2010). Results of many earlier functional studies have supported the notion that aberrant neural networks lie at the heart of ASD and obsessive–compulsive disorders (OCD). A core neurobiological mechanism that is putatively linked to ASD symptoms involves aberrant neural connectivity [see reviews Courchesne and Pierce (2005) and Wass (2011)]. Similarly, neurobiological models of OCD have been reported with a circuit involving orbitofronto-striato-thalamic systems (Remijnse et al., 2006; Chamberlain et al., 2008). Results of recent neuroimaging studies have demonstrated that more widely distributed large-scale brain systems are involved in OCD pathogenesis (Ping et al., 2013; Piras et al., 2015).

Electroconvulsive therapy (ECT) has been widely used for the treatment of many psychiatric disorders. Although the American Psychiatric Association task force on ECT states that ECT is not an effective treatment option for patients with OCD (Frankel, 1990), reports in the relevant literature have described ECT being effectively used in OCD (Goodman et al., 1993; D’Urso et al., 2012; Raveendranathan et al., 2012). Additionally, ECT is an effective alternative for treating coexisting acute phase of catatonia in ASD when benzodiazepine treatment proves to be insufficient (Zaw et al., 1999; Wachtel et al., 2008, 2010). Although the underlying neural mechanisms remain unclear, recent neuroimaging studies have supported the hypothesis that amelioration of functional connectivity in neural networks plays a key role in the working action of ECT (Fosse and Read, 2013).

Electroencephalography (EEG) provides a precise millisecond-timescale to examine normal and pathologic temporal dynamics. As reviewed by Billeci et al. (2013), many EEG analysis methods have been applied, revealing altered neural networks in ASD during both rest and specific task conditions using coherence or phase synchronization analysis.

Neural networks are characterized by dynamical neural communications in functionally specialized assemblies and long-range mutual interactions across these assemblies (Varela et al., 2001; Schnitzler and Gross, 2005; Sporns, 2011). Neurophysiologic output signals derived from EEG therefore exhibit complex temporal fluctuations that reflect non-linear dynamical processes (Tononi et al., 1998; Abarbanel and Rabinovich, 2001). The recent non-linear approaches to characterizing complex temporal dynamics have provided new insights into EEG dynamical complexity in mental disorders including ASD (Takahashi, 2013). One approach to the non-linear estimation of EEG complexity is entropy-based algorithm. Among entropy-based algorithms, sample entropy (SampEn) was developed as a practically tractable physiological complexity measure for evaluating signal regularity or predictability (Richman and Moorman, 2000). However, complexity that is inherent in physiological systems has a more restrictive concept: complex systems are neither completely regular nor absolutely random. Moreover, irregularity does not necessarily correlate with complexity (Tononi et al., 1998; Costa et al., 2005). Therefore, SampEn as a measure of irregularity does not imply intrinsic physiological complexity (Costa et al., 2002, 2005). Additionally, as current neural activity can be influenced by past neural processes that have been stored dynamically through feedback loops at multiple hierarchical levels of cortical processing (Fell et al., 2000), neurophysiologic signals might reflect historic effects on underlying dynamics at multiple temporal scales. To investigate the variation in physiological signals across multiple temporal scales, Costa et al. (2002) introduced multiscale entropy analysis (MSE), which is calculated based on SampEn, in recognition of the likelihood that the dynamical complexity of biological signals might operate across a range of temporal scales. Consequently, evaluating a particular pattern of entropy values across the varying scales permits the detection of intrinsic complexity. As a consequence, characterizing the EEG complexity using MSE might add another dimension to already identified neural dynamics of ASD [see a review by Billeci et al. (2013)].

We encountered a patient with ASD who, after ECT, exhibited dramatically improved coexisting severe catatonic obsessive–compulsive symptoms. This study examines changes in EEG complexity with ECT using MSE and its relation to clinical outcomes.

## Background

### Patient and case history

T., an 18-year-old man with ASD, came to our hospital complaining of overall movements slowing. At the age of 14 years, he was diagnosed with ASD according to the Autism Diagnostic Interview-Revised (ADI-R) based on the following developmental history. At age 2, he showed poor eye-contact and repetitive behaviors, avoiding group play. His repetitive interest was arranging his toys at equal intervals in a row by himself. He did not exhibit delayed language development. He had been unable to wear clothes other than made from soft material because of his hypersensitivity to tactile sensation. At age 6, he developed blinking tics and worsening of repetitive behaviors. He reportedly showed average intelligence on IQ tests (full IQ: 106). By the age of 17, his self-care behavior such as bathing and changing clothes had become slower. He also developed obsessive–compulsive symptoms. He had symmetry obsessions with ordering, arranging, counting compulsions, ruminations, dwelling on cosmology, and magical thinking, and tried to dispel them by performing repetitive rituals such as “rubbing the left ear and then the right elbow.” He was admitted to a hospital and was prescribed olanzapine. Nevertheless, his symptoms worsened. He would remain immobile for several hours and would show inability to initiate movements without prompting from others. Finally, he was transferred to our hospital, exhibiting catatonic features such as mutism, immobility, grimacing, negativism, posturing, and stereotypy.

His EEG and brain magnetic resonance imaging showed no abnormalities. Blood examinations including thyroid function yielded normal results. Neurological symptoms were also unremarkable. Escitalopram was started on the olanzapine and titrated up to 20 mg/day. His catatonic features improved slightly but remained severely impaired. Then, lorazepam was added up to 3 mg/day. He showed resolution of his catatonic features, which nevertheless brought him no relief. Because ECT is included in treatment algorithms for acute phase of catatonia in ASD (Fink et al., 2006), ECT was selected to gain catatonia remission. Lorazepam was discontinued to achieve appropriate ECT efficacy. His catatonic symptoms worsened again after stopping the lorazepam treatment. Other medications were maintained throughout the treatments. His response to ECT had been dramatic. He was making satisfactory progress, but he experienced severe headaches as an adverse effect of ECT. Generally, in spite of ECT’s favorable effect, its maintenance use is limited by the fact that ECT mostly appears to be beneficial in a temporary manner, except in a few cases (DeJong et al., 2014). Additionally, he had already demonstrated clinical relief from catatonic features. For these reasons, ECT was discontinued after a course of seven ECTs. Unfortunately, he relapsed slowly to obsessive–compulsive symptoms. Considering the fact that he was not that much more catatonic than before and that he had severe headaches with ECT, lorazepam (1.5 mg/day), to which he had responded moderately in the prior administration, was restarted. His problematic symptoms were alleviated. He became able to live independently. He was therefore discharged from our hospital. As of this writing, after no relapse for 5 months, he attends a day care center.

T. and his mother gave written informed consent to the publication of his case. We altered or omitted some details to avoid identifying the patient.

### Electroconvulsive therapy

This patient was naïve to ECT treatment. Bifrontal ECT was conducted (Thymatron System IV; Somatics LLC, Lake Bluff, IL, USA) twice a week. The seizure threshold for the first treatment and mean charge were, respectively, 151.1 and 85.8 mC. The mean seizure length was 63.29 s. Anesthesia was induced with thiopental 200–250 mg, succinylcholine 40–60 mg, and 100% oxygen. Table 1 presents ECT course details.

**Table 1:** **Electroconvulsive therapy (ECT) profiles**.

No. of ECT		1	2	3	4	5	6	7	
								1	2
ECT	Seizure threshold for ECT charge (mC)	30% (151.1)	25% (125.2)	20% (100.1)	15% (74.7)	10% (49.8)	10% (49.8)	5% (49.8)	10% (49.8)
	Seizure duration (s)	54	69	55	57	60	70	0	78
Anesthesia	Thiopental (mg)	200	200	200	200	200	200	200	200
	Succinylcholine (mg)	50	50	60	60	60	60	60	60

### EEG recordings

Electroencephalography activity was recorded with the subject lying in bed in a dark, soundproof laboratory room with no mental task. Standard scalp electrodes were placed in accordance with the International 10–20 System, referenced to linked earlobe electrodes. The EEG was recorded at a 500 Hz sample frequency with a 1.5–120 Hz bandpass filter using an 18-channel system (EEG-1214; Nihon Kohden Corp., Tokyo, Japan). Because of his hypersensitivity to tactile sensation, we selected eight electrodes (i.e., F3, F4, C3, C4, P3, P4, O1, and O2), which minimally cover the brain. Eye movements were monitored using additional electro-oculographic (EOG) channels. Impedances were maintained at <5 kΩ. Except for band pass filtering, no other pre-processing step (i.e., filtering, artifacts removal, or data reconstruction) was conducted because of the likelihood that such processing might distort non-linearity in the data, which might affect the MSE assessments. The state of vigilance was visually inspected with EEG traces during a recording session. When the subject appeared to be drowsy, he was asked to open his eyes and was verbally warned to avoid drowsiness. The selection of segments recorded during eyes-closed wakefulness was performed by visual inspection of EEG and EOG recordings. He was regarded as fully awake when alpha activity appeared predominantly over the posterior regions, with concurrent fast eye movements in the EOG channel (Wada et al., 1996). Additionally, spectral analysis was performed to confirm the appearance of alpha activity in O1 and O2 electrode sites. The EEG was recorded for 20–40 min to obtain two continuous resting state 30 s segments of data (15,000 data points: 30 s × 500 Hz). Segments with artifacts such as eye movements, blinks, muscle activities, and other artifacts were visually identified and were excluded. The EEG was recorded twice a week for a total of 24 times: pre-treatment (three times), during ECT (7 times), after ECT (8 times), and treatment with lorazepam (6 times). During ECT, EEG was performed 48 h after each ECT.

### Multiscale entropy

The MSE method quantifies the degree of irregularity in a time series at multiple time scales (Costa et al., 2005). First, to obtain multiple time scales, the original EEG time series {*x*_1_, *x*_2_, …, *x*_N_} was coarse-grained using the scale factor (SF) τ, with non-overlapping windows as shown below.

yjτ=(1τ)∑i=j−1τ+1jτxi,1≤j≤Nτ

Second, the irregularity of each coarse-grained EEG time series {*y*^(τ)^} was measured using SampEn, which is well suited for analyzing short and noisy experimental data (Richman and Moorman, 2000; Richman et al., 2004). SampEn is the negative of the logarithmic conditional probability that two sequences of *m* consecutive data points, which are mutually similar (within given tolerance *r*) will remain similar at the next point (*m* + 1) in the dataset (*N*), where *N* is the length of the time series. Considering the EEG time series {*x*_1_, *x*_2_, …, *x*_N_} as observations of a stochastic variable *x*, dynamic SampEn is defined as:

$$h_{\text{samp}}\left(r,m,N\right)=-\log_{e}\left[C_{m+1}\left(r\right)∕C_{m}\left(r\right)\right],$$

where Cm(r)={number of pairs (i,j)|zim−zjm|<r, i≠j}∕{number of all probable pairs, i.e.,(N−m+1) (N−m)}. Therein, *z* = *y*^(τ)^, and *z*^*m*^ is a vector of *m* sample time series of (*N − m*) length, and $\left|z_{i}^{m}-z_{j}^{m}\right|$ denotes the distance between points $z_{i}^{m}$ and $z_{j}^{m}$ in the space of dimension *m*, and *r* is the effective filter for measuring consistency of time series. For the coarse-grained time series at SF = 1, the time series {*y*^(1)^} was simply identical to the original time series. The SampEn values for low SFs captured short-range temporal irregularity, whereas higher SFs captured long-range temporal irregularity. Consequently, the SampEn values at smaller SFs were examined specifically for EEG complexity at high frequencies, whereas larger SFs were examined specifically at low frequencies. Various theoretical and clinical applications have shown that *m* = 1 or 2, and *r* = 0.1–0.25 of the SD of the data points provides good statistical validity for SampEn (Lake et al., 2002; Richman et al., 2004). For the present analyses, we used *m* = 2 and *r* = 0.2, which are values that were applied successfully in our previous study (Takahashi et al., 2009, 2010; Mizuno et al., 2010; Okazaki et al., 2013; Ueno et al., 2015).

To index information related to long-range temporal dynamics adequately, the EEG signals used for MSE analysis corresponded to continuous 30 s (15,000 data points), artifact-free segments selected from eye-closed resting condition, which are long compared to those used for other EEG analysis methods. For each subject, MSE was calculated on two segments and averaged into a single value. The MSE calculation was conducted with self-produced software, developed using a commercially available software package (Mathematica 8; Wolfram Research, Inc.). To examine the reproducibility of MSE results from two segments in the same EEG session, the Pearson product–moment correlation coefficients across ECT sessions were calculated. As a result, correlation coefficients were 0.87 for the frontal, 0.80 for the central, and 0.93 for the occipital region.

### Power spectrum analysis

**Figure 1:**
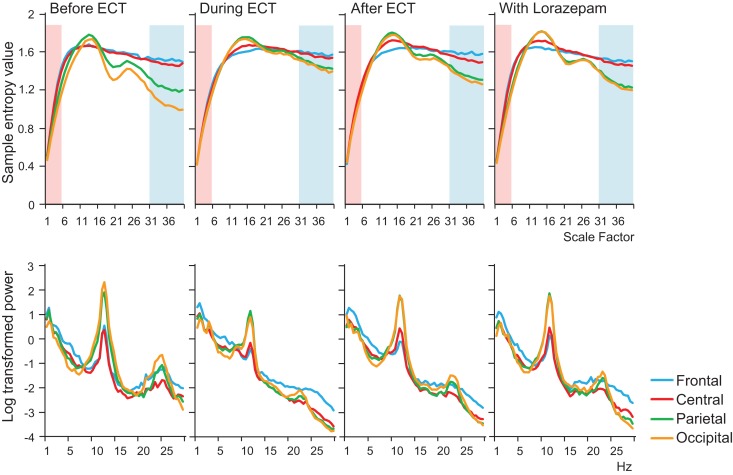
**Results of multiscale entropy (MSE) analysis (upper panel) and power spectrum analysis (lower panel) conducted before electroconvulsive therapy (ECT), during ECT, after ECT, and during treatment with lorazepam**. Each panel shows averaged MSE and power spectrum results from each condition. A decrease in sample entropy (SampEn) was observed with ECT at lower scale factors (SFs) 1–5 (highlighted with light pink shaded area). By contrast, increased SampEn was observed at higher SFs 31–40 (light blue shaded area).

In addition to MSE analysis, we performed power spectrum analysis as a comparative, more conventional EEG analysis, using a computer program (Brain Vision Analyzer 2; Brain Products GmbH, Germany). The spectral density was calculated using a fast Fourier transform (FFT). A Hanning window was applied to each 2 s epoch selected from 30 s artifact-fee segments that was also used for MSE calculation (i.e., a total segments for FFT was 15). In Figure 1, the absolute power spectrum values were log-transformed.

### Clinical assessment and brain-derived neurotrophic factor

His severe obsessive–compulsive symptoms presented catatonic features. Therefore, the Bush–Francis Catatonia Rating Scale (BFCRS; Bush et al., 1996) was administered for clinical assessments. We examined the serum concentrations of brain-derived neurotrophic factor (BDNF) because BDNF is a central part of the molecular hypothesis of ECT (Sartorius et al., 2009) and because it plays key roles in the pathogenesis of both ASD (Das, 2013) and OCD (Wang et al., 2011).

## Results

**Figure 2:**
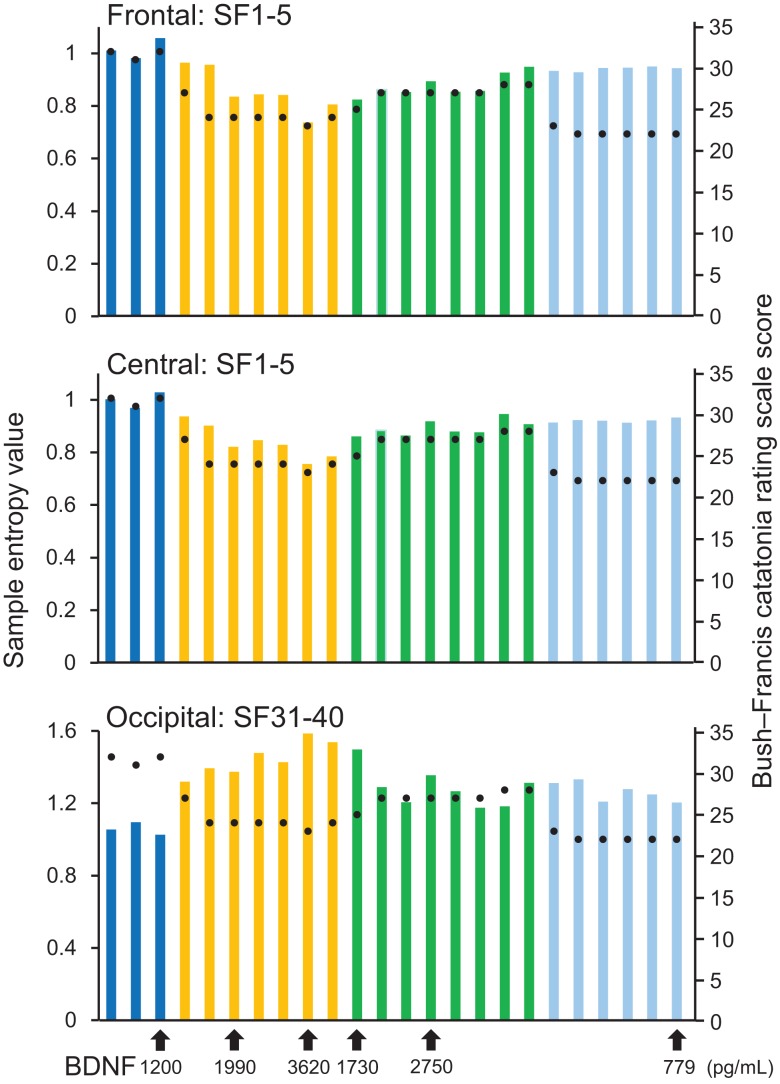
**Results from sample entropy (SampEn) values**. In the upper and middle panels, each bar shows averaged SampEn values of SF 1–5 (i.e., light pink shaded area in Figure 1) and for the frontal and central regions (F3, F4, C3, and C4). Each bar in the lower panel shows averaged SampEn values of SF 31–40 (i.e., light blue shaded area in Figure 1) for the occipital region (O1 and O2). Each condition was identified by color: before-ECT as the blue bar, during-ECT as the yellow bar, after-ECT as the green bar, and during treatment with lorazepam as the light blue bar. Regarding the clinical course, the black circle shows the Bush–Francis Catatonia Rating Scale (BFCRF). The vertical axis on the right shows the scale of the BFCRS score. The black arrow below the horizontal axis shows the serum concentration of BDNF.

**Figure 3:**
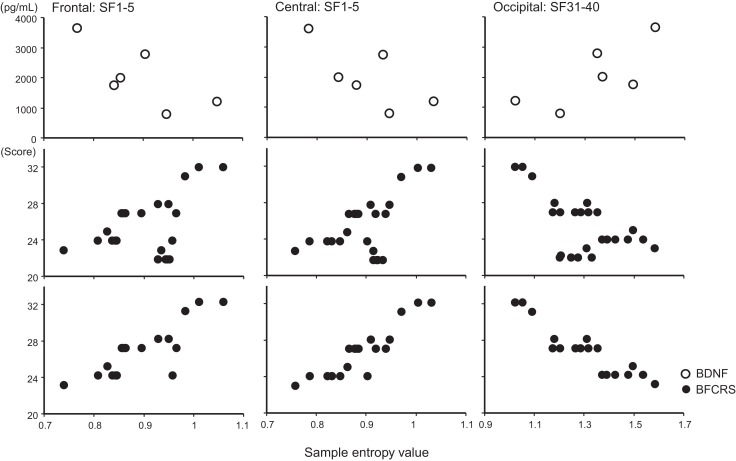
**Scatter plot associations of BDNF (top panel), BCRF (middle and bottom panel), and EEG complexity**. Compare middle and bottom panel; data obtained after treatment with lorazepam were eliminated from the middle panel.

A remarkable change of EEG complexity was observed in association with ECT treatment (Figure 1). At smaller SFs (i.e., light pink shaded area), the EEG complexity was decreased especially in the frontal and central regions. By contrast, at larger SFs (i.e., light blue shaded area), EEG complexity was increased especially in occipital regions. After ECT, these changes tend to revert to the level prevailing before treatment, but they remained, respectively, lower and higher. To clarify the possible association of the clinical course (BCRF and BDNF) with EEG complexity, we averaged SampEn values of SF 1–5 (i.e., light pink shaded area in Figure 1) for F3, F4, C3, and C4, and SF31–40 (i.e., light blue shaded area in Figure 1) for O1 and O2 into one value. Figure 2 presents a summary of the associations of changes in clinical course (BCRF and BDNF) to EEG complexity. In contrast to MSE, power spectrum analysis showed a power density decrease with ECT in the slow frequency range from theta to alpha, which was increased to the level of before treatment again, whereas the fast frequency range from beta to gamma showed a decrease with ECT, which tended to remain lower (Figure 1). This trend was observed for all electrode sites. Marked association is apparent between the EEG complexity and clinical improvement and BDNF level. However, the administration of lorazepam did not affect EEG complexity despite clinical improvement. Figure 3 presents scatter plot associations of clinical course (BCRF and BDNF) and EEG complexity (compare middle and bottom panel; data obtained after treatment with lorazepam were eliminated from the middle panel).

## Discussion

This report is the first describing ECT-induced change of the EEG complexity in a patient with ASD and coexisting obsessive–compulsive symptoms. Specifically, the frontocentral region showed a decrease in EEG complexity at lower SFs. However, the occipital region showed an increase at higher SFs. Our findings of increased and decreased EEG complexity in a region-specific and temporal scale-specific manner associated with clinical improvement and BDNF level might reflect atypical EEG dynamics in ASD.

A growing number of EEG and magnetoencephalography (MEG) reports describe abnormal neural connectivity with a region-specific and temporal scale-specific manner, and underpinned the concept of aberrant neural network in ASD [for a review, see Billeci et al. (2013)]. In terms of EEG–MSE study, Catarino et al. (2011) reported considerable reduction of EEG complexity in young subjects with ASD as the temporal scale increases, over temporoparietal and occipital regions. Additionally, reduced EEG complexity at higher temporal scales was reported in infants who were at high risk for autism, especially in the 9–24 months range, with 80% accuracy in discriminating high risk for autism subjects from normally developed infants (Bosl et al., 2011). Furthermore, region-specific and temporal scale-specific alterations were reported in patients with ASD in a resting-state MEG study (Ghanbari et al., 2015). Although our study examined only a single case and although it is limited by its lack of comparison with healthy controls, results described in these previous reports agree at least partially with our findings. This report describes a remarkable decrease of EEG complexity with ECT associated with clinical improvement in patients with depression, but this was apparent only at lower temporal scales (Okazaki et al., 2013). In this context, changes in EEG complexity at lower temporal scales in the frontal region might be due to the direct effects of ECT across mental diseases, although changes in the occipital region at higher temporal scales might be more specific to ASD’s neuropathophysiology, including obsessive–compulsive symptoms.

Potential limitations of our study must be considered. First, the influence of medications on MSE results must be considered because our patient had been consistently prescribed olanzapine and escitalopram during ECTs. In fact, we previously reported a significant EEG complexity decrease at higher temporal scales with typical antipsychotics in patients with schizophrenia, especially in the frontal region (Takahashi et al., 2010). Therefore, the possibility of psychotropic agent effects on EEG complexity must be discussed. However, medications of our case were maintained throughout the EEG examinations, except for lorazepam. We assume that the EEG complexity change should be a result of ECT. Second, the regional specificity described above must be discussed carefully. We examined only eight electrodes. Additionally, the electrical activity in each scalp electrode might not have its origin in the brain area directly underneath the electrode. Therefore, it is difficult to infer the precise spatial distribution of regional changes. Another important question remains: how is EEG complexity related to neural hyper-connectivity and hypo-connectivity? To elucidate the relation between EEG complexity to neuronal networks, Friston (1996) assessed the effect of the degree of neural connectivity within neuronal populations and measures of complexity using synthetic neuronal models, finding that reduced connectivity increases EEG signal complexity. Several reports in the literature have described investigations of neural connectivity and complexity. In healthy adolescents, Anokhin et al. (1999) investigated relations between global EEG dynamics and intelligence using EEG coherence and dimensional complexity, with the expectation that “coherence and dimensional complexity would be inversely correlated.” They found inverse correlation between coherence and dimensional complexity. In mental disorders, Jelles et al. (2008) investigate the relation between EEG coherence and correlation dimension in Alzheimer’s disease, and therefore, to gain further insight into the “disconnection” hypothesis of cognitive dysfunction in this disorder. The finding was that coherence and correlation dimensions are inversely correlated. Ghanbari et al. (2015) jointly examined MSE and synchronization likelihood to obtain a more detailed characterization of resting-state MEG activity in ASD. It is particularly interesting that group differences between ASD and typically developed subject in complexity and synchronization appear spatially complementary, such that where synchronization was elevated in ASD, complexity was reduced (and vice versa). In this sense, hyper-connectivity and low-complexity might be mutually related.

Taken together, the exact relation between complexity and the degree of connectivity remains unexplained and is open to debate, especially when discussing the complexity behavior and its relations to a spatial distribution of connectivity. Consequently, complementary techniques with better spatial resolution (e.g., MEG) and a direct measure of neural connectivity (e.g., mutual entropy or Omega-complexity) are necessary to obtain more precise inferences of complexity and connectivity. However, MSE analysis might provide additional insights into the role of specific neural dynamics, possibly reflecting the specific aberrant neural network activity, and the therapeutic neurophysiological mechanism of ECT in ASD. Furthermore, MSE might be useful as a benchmark of the therapeutic efficacy of ECT for patients with ASD.

## Conflict of Interest Statement

The authors declare that the research was conducted in the absence of any commercial or financial relationships that could be construed as a potential conflict of interest.

## Abbreviations

ASD, autism spectrum disorder; BDNF, brain-derived neurotrophic factor; BFCRS, Bush–Francis Catatonia Rating Scale; ECT, electroconvulsive therapy; EEG, electroencephalogram; MSE, multiscale entropy; OCD, obsessive–compulsive disorders; SampEn, sample entropy; SF, scale factor.
